# Small-Vessel Thrombotic Vasculopathy Secondary to Paradoxical Emboli Traversing a Patent Foramen Ovale: A Vasculitis Mimic

**DOI:** 10.1155/crrh/8840496

**Published:** 2025-02-26

**Authors:** Trevor Kwan, Steven Tu, Carlos El-Haddad

**Affiliations:** Department of Rheumatology, Liverpool Hospital, Liverpool, New South Wales, Australia

## Abstract

Paradoxical thromboembolism via intracardiac defects have been described to cause limb ischaemia by occluding medium- to large-vessels. No cases have described injury to only the small vessels of the feet. We present a case of a 20-year-old male presenting with painful dusky digits of both feet who was initially thought to have a small-vessel vasculitis, but instead found on histopathological examination to have acute thrombotic vasculopathy causing cutaneous ischaemia. He was subsequently found to have a patent foramen ovale (PFO) but no thrombosis elsewhere. This case underscores the importance of transthoracic echocardiography (TTE) in patients presenting with small-vessel ischaemia, even in the absence of deep venous thromboses.

## 1. Introduction

Venous thromboses paradoxically embolising to the systemic arterial circulation via an intracardiac defect, such as a patent foramen ovale (PFO), are a rare, yet recognised phenomenon [1]. Depending on the vascular territories affected, manifestations may include stroke, myocardial infarction, ischaemic bowel and acute limb ischaemia [1]. Reports of paradoxical emboli resulting in acute limb ischaemia have been described to affect the medium- to large-vessels of the limbs. However, to our knowledge, no cases have involved solely the microvasculature of the feet.

## 2. Case Presentation

**Figure 1:**
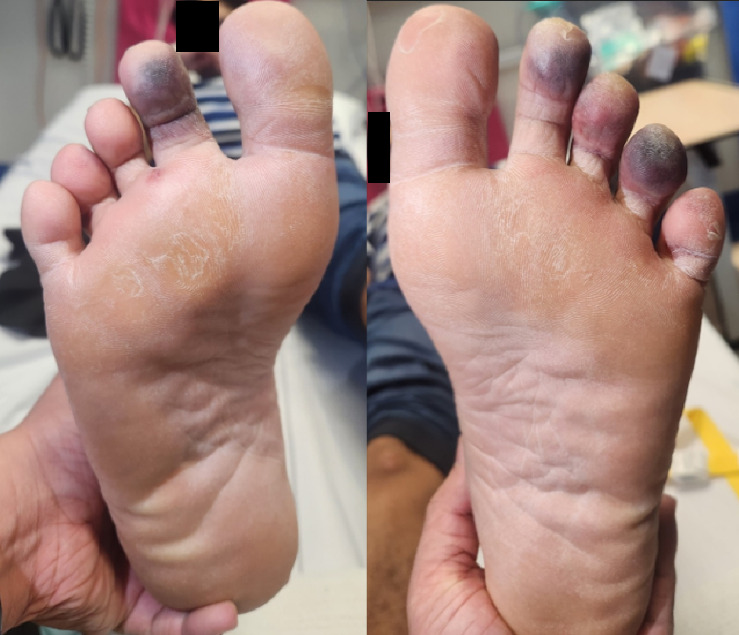
The plantar aspect of both feet on presentation.

**Figure 2:**
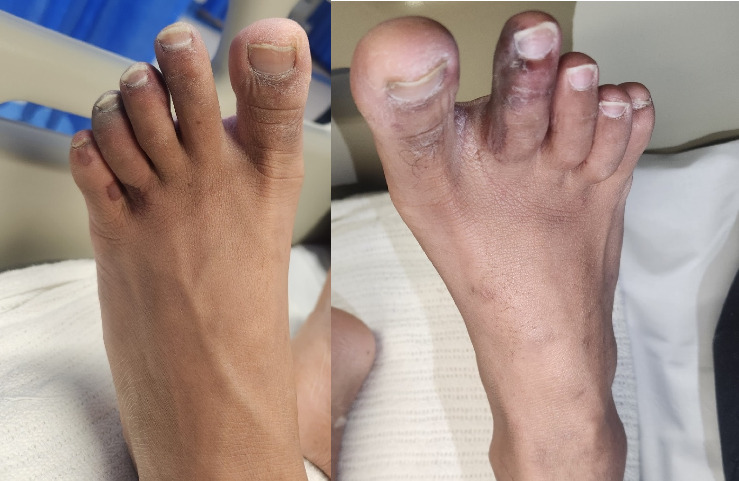
The dorsal aspect of both feet on presentation.

A 20-year-old male of Vietnamese heritage presented to Emergency with a 2-week history of parasthesias followed by duskiness and pain affecting digits of both feet (Figures 1 and 2). These changes occurred in the absence of preceding trauma or systemic symptoms such as fevers.

On examination, he was afebrile, and his other vital signs were unremarkable. He had strong popliteal, posterior tibial and dorsalis pedis pulses bilaterally. There were no calcinoses, Raynaud's, sclerodactyly or telangiectasias. There was no malar rash, synovitis, aphthous ulcers, or livedo reticularis. His heart sounds were dual, and there were no added sounds. His lung fields were clear. Nailfold capillaroscopy did not identify dilated capillary loops or dropouts.

### 2.1. Past Medical History

He had no past medical history of note and did not take any regular medications. He was a nonsmoker, nondrinker and had no history of recreational drug use. He had recently lost his job and spent most of his day sedentary at home.

### 2.2. Differential Diagnosis

The differential diagnoses included small-vessel vasculitis, connective tissue disease-related skin changes, drug-induced vasospasm and thromboembolic phenomena.

### 2.3. Investigations

Laboratory studies revealed a white cell count of 13.8 × 10^9^, a neutrophil count of 11.4 × 10^9^, an erythrocyte sedimentation rate of 21 mm/hour, a C-reactive protein of 7.1 mg/L, a creatinine of 97 μmol/L, a prothrombin time of 13.8 s and an activated partial thromboplastin time of 33 s. There were no other significant abnormalities on initial blood chemistry or full blood count. His electrocardiogram was in sinus rhythm.

Investigations for autoimmune vasculitic aetiologies, including antinuclear antibodies, antineutrophilic cytoplasmic antibodies, rheumatoid factor, and cryoglobulins returned negative. Infectious-associated vasculitides screen for human immunodeficiency virus, and hepatitis B and C, returned negative. Urine microscopy demonstrated < 10 × 10^6^/L red cells, a normal albumin to creatinine ratio of 0.6 mg/mmol, no dysmorphic red cells or casts and a urinary drug screen did not detect any illicit substances.

**Figure 3:**
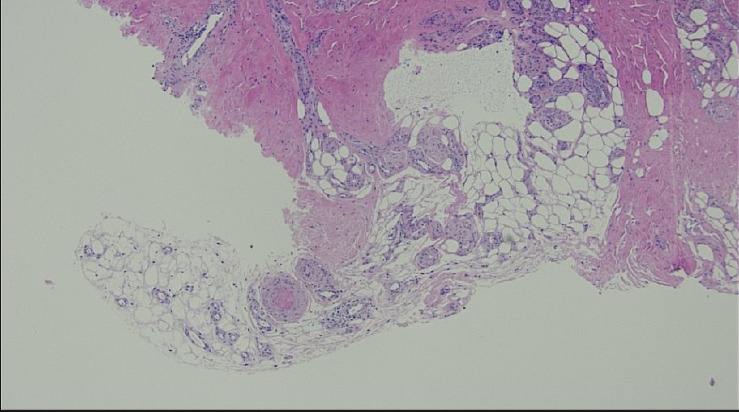
Superficial subcutaneous tissues with noninflammatory thrombus in a small vein (40x magnification).

**Figure 4:**
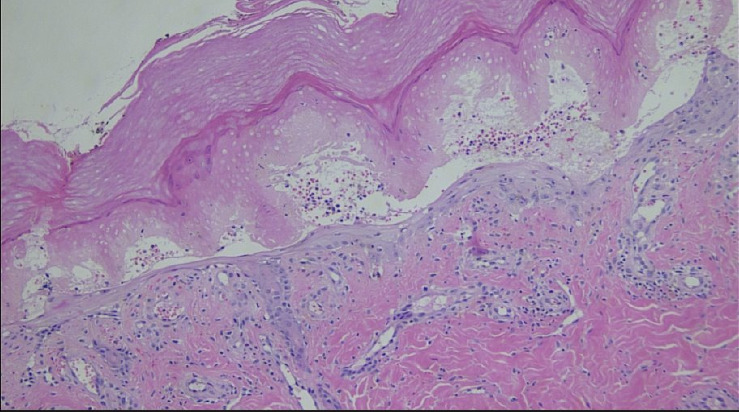
Necrotic epidermis and regeneration of attenuated squamous epithelium at the dermoepidermal junction (100x magnification).

**Figure 5:**
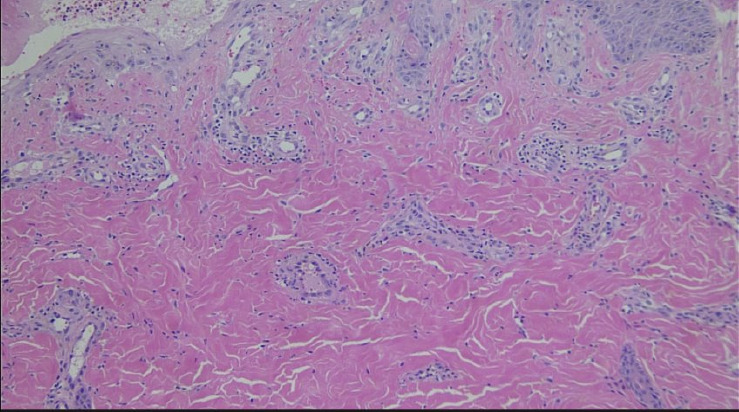
Vascular ectasia and mild perivascular lymphocytic infiltrate in dermis (100x magnification).

**Figure 6:**
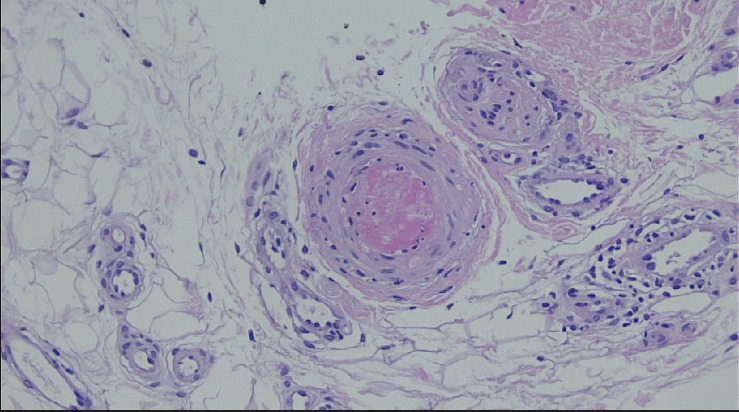
Thrombus occluding a small vein in the superficial cutis (200x magnification).

Vascular surgery was consulted and arranged for a duplex ultrasound of the aortoiliac and lower limb arterial segments which demonstrated normal flow characteristics, no sonographic evidence of acute arteritis and no adjacent deep venous thromboses. Dermatology was consulted and performed a punch biopsy of the skin overlying the left fifth digit of the foot. Histopathological examination of this sample demonstrated acute cutaneous ischaemia associated with noninflammatory thrombosis of the cutaneous vessels (Figures 3, 4, 5, and 6). No cholesterol clefts or vascular calcifications were seen.

He proceeded to undergo further thromboembolic workup. A computed tomography aortogram with delayed-phase distal run-off examining the popliteal arteries and beyond failed to demonstrate an intra-arterial thrombus, and there were three vessel run-offs bilaterally. A transthoracic echocardiogram (TTE) with an agitated saline contrast study demonstrated a right-to-left atrial level shunt. A subsequent transoesophageal echocardiogram with an agitated saline contrast study demonstrated a small PFO, but no intracardiac thrombus or valvular disease (Supporting Video 1).

Thrombophilia screening, including peripheral surface markers, serum electrophoresis and immunofixation, paroxysmal nocturnal haemoglobinuria screening, antithrombin III and protein C levels, antiphospholipid antibodies and JAK2 and Factor V Leiden gene mutations were normal. Protein S was mildly reduced at 55%; however, this was considered difficult to interpret in the context of an acute thrombotic event.

### 2.4. Management

On initial presentation, antiplatelet and vasodilator therapy was given. This included aspirin 100 mg daily and oral nifedipine sustained release 30 mg daily followed by intravenous iloprost 0.5–2.0 ng/kg/min over 6 h for 5 days with minimal improvement in his symptoms. Following the biopsy results, vasodilator therapy was stopped. Aspirin was ceased and changed to oral rivaroxaban 20 mg daily in consultation with a cardiologist.

### 2.5. Outcome and Follow-Up

He is planned for follow-up with a structural cardiologist to organise closure of his PFO.

## 3. Discussion

PFOs can be seen in approximately 25% of the adult population [1, 2]. A majority of these are clinically silent; however, a minority may be considered clinically significant when they are associated with a paradoxical venous thromboembolism affecting the systemic circulation [1, 2]. Under physiological conditions, PFOs are closed passively due to the positive pressure gradient across the left to right atria [1]. PFOs may be opened when right atrial pressures rise, causing a reversal in the pressure gradient and a right-to-left shunt [1]. This can occur physiologically, such as at the conclusion of Valsalva manoeuvres, or pathologically, such as in pulmonary hypertension [1].

Paradoxical embolism is a challenging diagnosis that requires a high index of suspicion to prompt further investigation. Although there are no established diagnostic criteria, it is generally accepted that a diagnosis can be made if there is an intracardiac defect or pulmonary shunt, as well as evidence of an arterial embolism [1, 2]. As the primary thrombus is thought to originate from the deep venous systems, they tend to be larger and occlude larger vessels when embolising to the lower extremities [3]. It was therefore unusual to encounter a case where no thrombus was seen in any of the medium- and large-vessels of the lower limbs on duplex ultrasonography and computed tomography angiography. Deep venous thromboses are supportive of the diagnosis but are infrequently detected [2].

Considering the diagnostic findings, the overall confidence that the acute thrombotic vasculopathy was due to a paradoxical embolus was high. It was therefore felt that anticoagulation should be initiated prior to the planned investigation for deep venous thrombosis. Considering the negative thrombophilia screen, it was speculated that the period of prolonged immobility was the precipitating event leading to the pathogenesis of a deep venous thrombosis.

Some parallels can be drawn between this case and occlusive vasculopathy, colloquially known as blue toe syndrome. Blue toe syndrome is an occlusive small vessel vasculopathy caused by cholesterol crystals or arterial atherothrombotic embolisation [4]. This syndrome presents most commonly in elderly men who have undergone angiography, vascular surgery or initiation of anticoagulation or thrombolysis [4]. Palpable pedal pulses are often a distractor from the vascular pathology, therefore leading to frequent misdiagnosis at presentation [4]. Vasospasm is usually absent, and hence vasodilatory treatment often confers minimal benefit [4]. In the absence of appropriate milieu, it is felt to be less likely that the emboli encountered in our case study were derived from the same pathophysiological processes as blue toe syndrome, despite causing a similar pattern of injury.

The optimal treatment strategy for paradoxical embolism is unclear, owing to the rarity of the condition and the lack of longitudinal data of patients treated following paradoxical embolism [5, 6]. Life-threatening, multiorgan paradoxical emboli have successfully been treated with prompt thrombolysis [7]. Other accepted medical therapies include a combination of antiplatelets and/or anticoagulants based on an individualised case-by-case assessment [5, 6]. Percutaneous PFO closure in combination with medical therapy is conditionally recommended in persons with systemic embolism, and without a prior PFO-associated stroke, in whom other embolic aetiologies have been excluded [8].

## 4. Conclusions

Paradoxical embolisation can rarely cause isolated small-vessel occlusive vasculopathy, masquerading as a small-vessel vasculitis. Investigating for paradoxical embolisation is well established in patients with cryptogenic stroke or large-vessel ischaemia. However, this case highlights the importance of TTE in patients presenting with small-vessel ischaemic changes, even in the absence of deep venous thrombosis. Early recognition of clinically significant PFOs is paramount, as timely anticoagulation may prevent potential fatal future events.

## Data Availability

All data related to this study are included in the report, and further enquiries can be directed to the corresponding author.

## Supporting Information

Supporting Video 1 is a video of our patient's transoesophageal echocardiogram which demonstrated a small patent foramen ovale with a positive agitated saline study.
